# Idiopathic pulmonary fibrosis in Sweden: report from the first year of activity of the Swedish IPF-Registry

**DOI:** 10.3402/ecrj.v3.31090

**Published:** 2016-04-21

**Authors:** Giovanni Ferrara, Lisa Carlson, Andreas Palm, Jonas Einarsson, Cecilia Olivesten, Magnus Sköld, for the Swedish Idiopathic Pulmonary Fibrosis Registry Group

**Affiliations:** 1 Department of Respiratory Medicine and Allergy, Karolinska University Hospital, Stockholm, Sweden; 2 Respiratory Medicine Unit, Department of Medicine, Solna and Center for Molecular Medicine, Karolinska Institutet, Stockholm, Sweden; 3 Gävle Hospital, Gävle, Sweden; 4 Skåne University Hospital, Malmö, Sweden; 5 Department of Respiratory Medicine and Allergology, Skåne University Hospital, Lund University, Lund, Sweden; 6 Sunderby Hospital, Luleå, Sweden

**Keywords:** idiopathic pulmonary fibrosis, registry, quality of life, K-BILD, lung function

## Abstract

**Background:**

Idiopathic pulmonary fibrosis (IPF) is an emerging problem in the western world, being related to increasing age and implying significant costs for the diagnosis and management of affected patients. The epidemiology of IPF is not well understood.

**Methods:**

To allow estimates of the problem and eventually to evaluate quality of the care of IPF patients in Sweden, a national IPF Registry was started in the autumn of 2014. Data on criteria used to diagnose IPF, demographics, lung function, and quality of life (measured with the King's Brief Interstitial Lung Disease Questionnaire, K-BILD) were reported directly to the registry, based at the coordinating centre (Karolinska University Hospital, Stockholm, Sweden) via a web-based platform.

**Results:**

During the first year, the registry was implemented in 11 (33%) of the 33 respiratory units in the country. Seventy-one patients were registered between October 2014 and October 2015, 50 (70.4%) males and 21 (29.6%) females. Median age was 70 (range 47–86). The mean K-BILD score at the first inclusion in the registry was 54.3+9.5.

**Conclusions:**

The main features of IPF patients in this first Swedish cohort were consistent with data published in the literature in main multinational randomized controlled trials. The K-BILD questionnaire showed that quality of life of patients with IPF and their perception of the disease are quite poor at the time of inclusion in the registry.

Idiopathic pulmonary fibrosis (IPF) is defined as a specific form of chronic, progressive fibrosis of unknown cause, occurring primarily in older adults, and limited to the lungs, with a survival of about 3–5 years from the diagnosis (1). A multidisciplinary approach involving specialists with expertise in the field is necessary to differentiate IPF from the other interstitial lung diseases (ILDs) (2). Estimates on IPF prevalence rates are derived from studies carried out with different methodologies mainly in the United States (3), Mexico (4), and England (5), and are still unknown in most of the countries around the world, including Sweden. A national Finnish study estimated the prevalence in Finland around 14–16/100,000 cases (6). Considering the lowest incidence estimates, as many as 432 new cases of IPF should be diagnosed in Sweden every year, with a prevalence of 1,000–1,500 cases.

The Swedish Guidelines for IPF (7), published by a national group of experts from the Swedish Respiratory Society (Svensk Lungmedicinsk Förening, or SLMF), are a first step to uniform and increase the standard of care for patients with IPF in Sweden. Its recommendations are in agreement with major international documents (1).

Two drugs (pirfenidone and nintedanib) are now registered based on large randomised clinical trials (8–11) and are commercially available in Europe and the United States. The high costs (estimated between 28,000 and 38,000 euros/year/patient) (12) and the relatively weak evidence concerning their effect on hard outcomes as mortality (13) have already fuelled a debate on how resources for patients with IPF should be allocated within the health-care systems.

To provide a platform to address these questions, a national quality registry for IPF was started in Sweden in September 2014.

The main objective of this paper is to demonstrate the feasibility of the implementation of a web-based IPF registry in Sweden and to report the results of the first year of activity of the Swedish IPF-Registry.

## Methods

A web-based platform, already in use in Finland (Granitics Unify Med, Granitics Ltd, Espoo, Finland), was chosen and adapted to Swedish healthcare and regulatory standards. The platform allowed the registration of demographics, lung function, radiology, quality of life (assessed with the King's Brief Interstitial Lung Disease Questionnaire (K-BILD)) (14), ongoing treatments, adverse events according to the Common Terminology Criteria for Adverse Events (15), follow-up and outcomes such as death and lung transplantation at the time of inclusion in the registry and during the follow-up. K-BILD was translated from English to Swedish for the purpose of being included in the registry (16). The K-BILD score measures quality of life on a scale from 0 to 100, with 100=best health status (14).

To be included in the registry, patients had to fulfil the diagnostic criteria for the diagnosis of IPF according to national and international guidelines (1–7). Table 1 illustrates all the variables collected in the Swedish IPF registry. A registered nurse was hired as registry coordinator with the task to establish contact with other hospitals and to provide on-site training and technical support.

**Table 1:** The Swedish Idiopathic Pulmonary Fibrosis Registry variables

Demographics	Gender
	Date of birth
	Age
	Reference hospital
Diagnosis (IPF)	Time of diagnosis
	ICD-10
	Base for diagnosis: - Clinical–radiological - Biopsy-proven - Based on MDC
Clinical	Height, Weight, BMI
information	Smoking status: - Ex-smoker - Current smoker - Never smoker
	Pack-year
	Profession, professional/environmental exposure
	Chronic comorbidities
	Ongoing treatments
	Main symptoms and time of onset
	Clinical findings: - Presence of gastric reflux - Drumstick fingers - Crackles at auscultation
Lung function	Lung plethysmography: FEV1, FEV1%, FVC, FVC%, FEV1/FVC, TLC, TLC%
	Diffusion capacity: DlCO%
	Pulse oximetry at rest; arterial blood gas analysis
	6MWT: saturation at rest and lowest value during/after the test; walk distance
Radiology	Chest X-ray
	HR CT: - UIP pattern - Possible UIP pattern - Inconsistent with UIP pattern
Pathology	BAL: total cell count, cell differentials
	Lung biopsy (open or VATS): - UIP pattern - Probable UIP-pattern - Possible UIP-pattern - Not UIP-pattern
Quality of life	K-BILD questionnaire
IPF therapy	Date of start, date of stop
	IPF specific therapies: - Pirfenidone - Nintedanib - NAC - Triple therapy
	Best supportive therapy: - Long-term oxygen therapy - Physiotherapy - Psychological support - Palliation (morphine, benzodiazepine)
	Adverse events: - Date of onset - Type of AE - Severity (according to CTCAE) - Intervention - Suspension/modification of the therapy
Outcome	Death: - Date of death - Cause of death
	Transplantation: - Date of transplantation
	Follow-up: - Lung function testing - Radiology - Quality of life (K-BILD) - Ongoing treatments/AEs/modification of IPF-therapies

IPF: idiopathic pulmonary fibrosis; ICD-10: International Statistical Classification of Diseases and Related Health Problems – Tenth Revision; MDC: multi-disciplinary conference; BMI: body mass index; FEV1: forced expiratory volume in 1 s; FEV1%: forced expiratory volume in 1 s, % of the expected value; FVC: forced volume capacity; FVC%: forced volume capacity, % of the expected value; TLC: total lung capacity; TLC%: total lung capacity, % of the expected value; DlCO%: Diffusion capacity of carbon monoxide, % of the expected value; 6MWT: 6-min walking test; HR CT: high-resolution computed tomography; UIP: usual interstitial pneumonia; BAL: bronchoalveolar lavage; VATS: video-assisted thoracoscopy; K-BILD: King's Brief Interstitial Lung Disease Questionnaire; NAC: *N*-acetyl-cysteine; AE: adverse event; CTCAE: Common Terminology Criteria for Adverse Events.

A cross-sectional analysis of the data reported at the time of inclusion in the registry between September 2014 and October 2015 is presented. Continuous variables are presented as mean±standard deviation of the mean or median and range. Categorical variables are presented as proportions of the total population. Single sample, paired *t*-test was used when appropriate, with level of significance at *p*<0.05.

The study was approved in August 2014 by the Stockholm's Regional Ethical Committee (Ref. No. 2014/1202-31/4). All patients needed to be informed by the case manager at his/her hospital about the aim of the project and to sign a written consent before inclusion in the registry.

## Results

The web-based platform was activated at the Karolinska University Hospital, Stockholm, Sweden, and made available for all participating centres in September 2014. The Karolinska University Hospital assumed the role of leading and legally responsible institution for the implementation of the project.

The initiative was made public with annual presentations at the Swedish Respiratory Society Congresses in Malmoe (2014) (17) and in Goteborg (2015) (18) and with thematic articles on IPF and the need of a national registry on the journal of the Swedish Respiratory Society (19, 20) and on local newspapers (21). A web page on the Swedish IPF-Registry was also published on the website of the Swedish Respiratory Society (22).

**Fig. 1:**
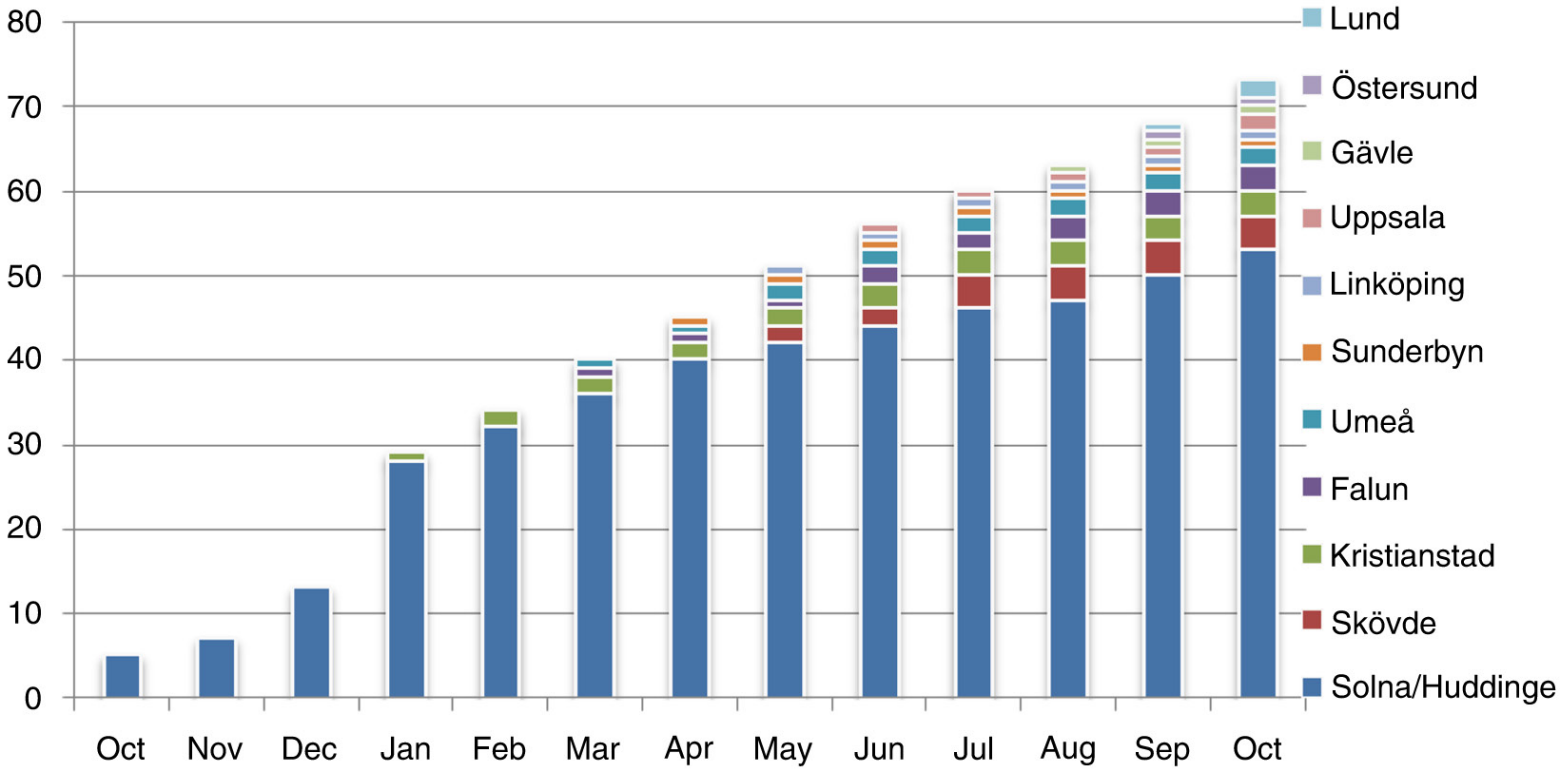
The distribution of the IPF-patients recorded in the Swedish IPF-Registry between October 2014 and October 2015 according to month (*x*-axis) and participating centre (*y*-axis).

During the study period, 14 of the 33 (42%) Swedish respiratory disease units were connected to the registry and 11 (33%) started to report data from patients with IPF in the first year of activity of the Swedish IPF-Registry. The hospitals participating in the data collection and the relative number of patients registered for each centre during the study period are reported in Fig. 1.

A total of 71 patients with IPF were recorded in the Swedish IPF-Registry during the first year of activity. Demographics and main features of the patients at the time of registration in the IPF-registry are reported in Table 2.

**Table 2:** Demographics and main clinical features of the patients included in the Swedish Idiopathic Pulmonary Fibrosis Registry

	Men	Women	Total
Patients (*N*, %)	50 (70.4%)	21 (29.6%)	71 (100)
Age (Median, range)	70 (47–86)	68.5 (51–84)	70 (47–86)
Smoking (*N*, %)	Never smokers: 12 (24%) Ex-smokers: 30 (60%) Current smokers: 0 (0%) Missing info: 8 (16%)	Never smokers: 8 (38.1%) Ex-smokers: 10 (47.6%) Current smokers: 3 (14.3%)	Never smokers: 20 (28.2%) Ex-smokers: 40 (56.4%) Current smokers: 3 (4%) Missing info: 8 (11.4%)
Pack-years among ex-smokers (M±SD)	24.5±14	11±11.6	20.7±14.6
BMI (M±SD)	26.8±3.3	27.8±6.8	27±4.5
Reflux (*N*, %)	10 (20%)	4 (19%)	14 (19.8%)
FEV1% of predicted (M±SD)	77.5±16.9	77.5±19	77.5±17.3
FVC% of predicted (M±SD)	70.2±15	77.4±20.4	72.3±16.9
FEV1/FVC (M±SD)	82.2±5.8	80±6.2	81.5±6
TLC, % of predicted (M±SD)	64.5±14	62.2±12.2	70.6±17.3
DlCO, % of predicted (M±SD)	50.9±16.9	54.5±19.4	52.1±17.6

M, mean; SD, standard deviation; BMI, body mass index; FEV1%, forced expiratory volume in 1 s; FVC, forced vital capacity; TLC: total lung capacity; DlCO%: diffusing capacity of the lung for carbon monoxide.

The diagnosis of IPF was based on clinical–radiological features in 52 (72%) patients, while 10 (14%) underwent a lung biopsy and 14 (20%) received a diagnosis after a multidisciplinary conference.

Thirty-one (44%) of the patients underwent a 6-min walking test at the time of inclusion in the registry; most of them presented a much lower SaO_2_ during or just after the test compared to the value at rest (95±0.9% at rest vs. 86±2.3% during the test, *p*<0.05).

The mean value of the K-BILD score was 54.3±9.5, demonstrating a relatively low quality of life at the time of inclusion in the registry.

## Discussion

Our report shows that web-based tools can rapidly be implemented and used to address important questions in healthcare at national level. Our experience reinforces previous reports from Australia, showing that technological tools can greatly improve the understanding of rare disease at a national level and overcome quite easily geographical distances and logistic problems (23).

The implementation process is crucial to the success of the project, and many practical and technical issues need to be promptly addressed while connecting the participating centres: to secure that data are correctly entered and timely reported is important to secure accuracy and completeness of the registry; on the other hand, the staff of the respiratory units involved in the project had already a clinical burden of everyday chores to perform in their hospitals. Part of the success of the initial implementation of the Swedish IPF-Registry was to identify committed doctors and motivated staff in the respiratory units. Support of a registry coordinator making regular visits to the participating centres to solve technical and practical issues was crucial to ensure engagement of the users and registered data of high quality. Users were constantly involved in a discussion on how to ease the registration of data, and the national guidelines on IPF served as a valuable source of definitions. Technical, IT, and practical issues (e.g. log in with staff ID, substitution of free text with checklist variables and tips on how to look fast through the journals to collect data) were continuously discussed with the registry coordinator and addressed with the IT company.

The patient cohort presented in this manuscript cannot be considered representative of the whole population of IPF patients in Sweden. Data on the epidemiology of IPF in Sweden are simply not available. One of the aims of starting an IPF-registry was in fact to provide estimates on this disease in Sweden, but this will be possible only if the registry covers at least 80% of the country. This goal might be reasonably reached in 2018.

The main features of the patients recorded in the Swedish IPF-Registry seem to resemble the clinical presentation of IPF as reported in multinational multicentre randomised controlled trials (8–11).

Males seem to be more affected than females, as shown in previous studies, as well as the high prevalence of ex-smokers among IPF-patients (24).

The diagnosis was mostly based on clinical–radiological features (according to the criteria identified by national and international guidelines) (1, 7), and only 10% of the patients underwent invasive procedures like lung biopsy in the diagnostic work-out. This is consistent with the data reported in the literature (25).

Interestingly, most of our patients were affected by a mild or moderate form of IPF according to the forced vital capacity (FVC) value. Nevertheless, most of them reported a relatively poor quality of life, with a mean K-BILD of 54.8; this finding supports an ongoing discussion questioning the value of FVC in the assessment and follow-up of patients with IPF. Perhaps total lung capacity and diffusion capacity for carbon monoxide might better correlate to quality of life, but further studies on large cohorts are needed to confirm this hypothesis. Data from the Australian IPF registry, presented at the latest European Respiratory Society Congress in Amsterdam in September 2015, showed no correlation between the decrease of FVC and the changes in quality of life recorded with the Saint George Respiratory Questionnaire (26). Larger numbers of patients and prospective longitudinal analysis are needed to assess which clinical parameters and outcomes correlate best with quality of life and disease perception/limitation in daily life.

## Acknowledgements

Thanks to a grant from the Swedish Heart and Lung Foundation (Ref. No. 20130706). We thank the Karolinska University Hospital, the Karolinska Institutet and the Quality-Registry-Centre Stockholm (QRC-Stockholm) for the technical support, and the Department of Respiratory Medicine and Allergy at the Karolinska University Hospital for supporting the project by providing resources to hire the registry coordinator. Boheringer Ingelheim contributed with a supporting grant in 2015 and Intermune/Roche supported the translation in Swedish of the K-BILD questionnaire. We thank Dr. Surrinder Birring for allowing the use of K-BILD and for the technical help he offered in implementing K-BILD in our registry. A special thanks to Helène Blomqvist, Margitha Dahl, Gunnel de Forest, Henrik Ryftenius, and Lise-Lotte Ladenfelt Gestrè for their help in the implementation of the registry at the headquarter of Karolinska University Hospital, Solna, Sweden.

**The Swedish Idiopathic Pulmonary Fibrosis Registry Group**: *Stockholm:* Giovanni Ferrara, Magnus Sköld, Lisa Carlson, Maria Diakopoulou, Valentyna Yasinska, Henrik Ryftenius, Lise-Lott Landenfelt Gestré, Gunnel de Forest, Margitha Dahl, Helene Blomqvist, Ingrid Gerhardsson, Olov Andersson. *Göteborg:* Anders Thylen, Kärstin Byström; *Umeå*: Kenneth Nilsson, Lena Granbom, Ala Muala. *Uppsala*: Shumi Omar, Carl-Axel Karlsson, Inger Dahlen. *Gävle:* Andreas Palm, Anna Svensson, Kristina Forsberg, Anna Bergengren, Carl Blomberg, Eva Branden, Johan Isaksson, Hirsh Koyi, Johanna Roos, Stefan Soneberg. *Falun*: Pierre Sobrino, Anders Pettersson, Anders Birkehag, Jon Goenechea, Wolfgang Greger, Gabriel Lundberg, Saba Raouf. *Helsingborg:* Rolf Rosin, Charlotta Berling, Lars Danielsson, Lena Eldh, Mats Lagerstedt. *Lund:* Jonas Einarsson, Helena Jonsson, Hillevi Larsson. *Linköping:* Lennart Persson, Ewa Petterstedt, Christel Bergström, Antje Kuhlman, Maria Sege. *Sunderby:* Dirk Albrecht, Christos Belias, Cecilia Olivesten. *Östersund:* Per-Olof Rydström, Nikolai Stenfors, Cristina Cretu, Jan Starlander. *Örebro:* Lennart Nilholm, Josefin Sundh. *Kristianstad:* Lars Andersson, Håkan Leksell, Anna Collen Kandell. *Skövde:* Anders Planck, Synnöve Alves, Gudrun Hemeren, Åke Johansson, Savvas Papadopoulos, Akos Somoskovi, Per Torstensson. *Trollhättan:* Bo Pedersen, Lars Johansson, Ulla Waxne, Milena Ymefors.
